# Homoarginine and methylarginines independently predict long-term outcome in patients presenting with suspicion of venous thromboembolism

**DOI:** 10.1038/s41598-021-88986-y

**Published:** 2021-05-05

**Authors:** Roman N. Rodionov, Jan Beyer-Westendorf, Stefanie M. Bode-Böger, Lisa Eggebrecht, Stavros Konstantinides, Jens Martens-Lobenhoffer, Markus Nagler, Jürgen Prochaska, Philipp Wild

**Affiliations:** 1 Division of Angiology, Department of Internal Medicine III, University Center for Vascular Medicine, University Hospital Carl Gustav Carus, Technische Universität Dresden, Fetscherstraße 74, 01307 Dresden, Germany; 2 College of Medicine and Public Health, Flinders University and Flinders Medical Centre, Adelaide, Australia; 3 Division of Hematology, Hemostaseology and Coagulation, Department of Medicine I, University Hospital Carl Gustav Carus, Technische Universität Dresden, Dresden, Germany; 4 Kings Thrombosis Service, Department of Hematology, Kings College London, London, UK; 5 Institute of Clinical Pharmacology, Otto-von-Guericke University, Magdeburg, Germany; 6 Department of Preventive Cardiology and Preventive Medicine, Center for Cardiology, University Medical Center of the Johannes Gutenberg University Mainz, Langenbeckstr. 1, 55131 Mainz, Germany; 7 Clinical Epidemiology and Systems Medicine, Center for Thrombosis and Hemostasis, University Medical Center of the Johannes Gutenberg University Mainz, Langenbeckstr. 1, 55131 Mainz, Germany; 8 Department of Cardiology, Democritus University of Thrace, University General Hospital, Alexandroupoli, Greece; 9 German Center for Cardiovascular Research (DZHK), Partner Site Rhine-Main, Mainz, Germany

**Keywords:** Biomarkers, Molecular medicine

## Abstract

Endogenous arginine derivatives homoarginine, asymmetric dimethylarginine (ADMA) and symmetric dimethyarginine (SDMA) are independent mortality predictors in atherosclerotic cardiovascular disease (CVD). Our study reports the first analysis, whether homoarginine, ADMA and SDMA predict venous thromboembolism (VTE) recurrence and overall mortality in patients with suspected acute VTE. We assessed serum levels of homoarginine, ADMA and SDMA by LC–MS/MS in 865 individuals from a prospective consecutive cohort of patients with clinical suspicion of VTE. The median follow-up time for mortality was 1196 days. VTE was confirmed by imaging in 418 patients and excluded in 447 patients. Low levels of homoarginine and high levels of ADMA or SDMA independently predicted all-cause mortality after adjustment for sex, age, oral anticoagulants, body mass index, arterial hypertension, diabetes mellitus, smoking, dyslipidemia, chronic heart failure, history of stroke, creatinine and cancer both in patients with VTE and without VTE. Interestingly, none of those parameters was predictive for VTE recurrence. We provide the first report that low circulating levels of homoarginine and high circulating levels of ADMA and SDMA independently predict all-cause mortality in patients with suspected VTE. These parameters might serve as markers of “frailty” and should be considered for future risk stratification approaches in this clinical population. Taking into account that homoarginine supplementation is protective in animal models of CVD and safe in healthy human volunteers, our study provides the basis for future homoarginine supplementation studies in patients with suspected VTE to investigate possible direct protective effects of homoarginine in this population.

## Introduction

Despite modern advances in diagnostics and therapy, venous thromboembolism (VTE), which encompasses deep vein thrombosis (DVT), pulmonary embolism (PE) or both, remains a major medical and socioeconomical challenge worldwide^1,2^. The annual incidence rate of VTE is about 1.5 in 1000 people and its lifetime prevalence is approximately 5%^3,4^. According to a recent study, in Europe in the years 2013–2015 pulmonary embolism accounted for 8–13 per 1000 deaths in women and 2–7 per 1000 deaths in men among individuals aged 15–55 years^5^. The main cause of death in VTE is right heart failure due to pulmonary embolism^6^. Although tools are available to categorize VTE patients according to their short term mortality risk, overall mortality and morbidity of this condition remain high, indicating an unmet clinical need for further optimization of the risk prediction tools. With this, patients at high short- and long-term risk for VTE complications and death could be identified, leading to a more individualized treatment for improved outcomes.

Endogenous analogues of L-arginine, such as asymmetric dimethylarginine (ADMA) and symmetric dimethylarginine (SDMA) and homoarginine have been proposed in clinical studies as markers and possible mediators of cardiovascular damage^7,8^. Association between elevated levels of ADMA and adverse cardiovascular outcomes might be at least partially explained by direct inhibitory effects of ADMA on nitric oxide (NO) production by NO synthases^9^. Furthermore, ADMA can cause eNOS uncoupling in the endothelial cells leading to increased superoxide generation^10^. Even though SDMA is not able to inhibit NO synthases, it may indirectly influence NO homeostasis by interfering with the transport of the NO precursor L-arginine^11^. In contrast to the dimethylarginines ADMA and SDMA, the correlation between homoarginine levels and cardiovascular risk is inverse, suggesting that endogenous homoarginine may serve a protective role^12–14^. Indeed, homoarginine supplementation was shown to be protective in experimental murine model of stroke, while the circulating homoarginine levels were inversely associated with fibrinogen, β-thrombomodulin, and von Willebrand factor in patients with stroke^13^. Prospective LURIC (LUdwigshafen RIsk and Cardiovascular Health) study showed inverse associations of homoarginine with fibrinogen and D-Dimers in patients referred to coronary angiography, thus further supporting potential role of low homoarginine levels as a prothrombotic marker^12^. The mechanism, however, is not known.

While the predictive value of elevated levels of dimethylarginines ADMA and SDMA and low levels of homoarginine in the arterial and myocardial cardiovascular diseases has recently been established^15^, the role of these molecules as outcome markers in VTE is currently unknown. The goal of this study was to test the hypothesis that high levels of endogenous dimethylarginines ADMA and SDMA and low levels of homoarginine predict VTE recurrence and overall mortality in patients with acute VTE.

## Material and methods

### Data availability

The authors declare that all the supporting data are available within the article. Unprocessed data are available from the corresponding authors on request.

### Study design

Overall, 865 individuals from the VTEval cohort^16^ encompassing patients presenting with clinical suspicion for DVT (551 patients), clinical suspicion for PE (295 patients) or with established incidental (asymptomatic) VTE in imaging procedures (19 patients) were included into this study. The study was performed according to the principles of good clinical practice and the principles outlined in the Declaration of Helsinki; approval by the local ethics committee (medical association of the federal state Rhineland-Palatinate, Germany; reference no. 837.320.12 (8421-F)) and the data safety commissioner of the University Medical Center of the Johannes Gutenberg University Mainz was obtained prior to study initiation. Written informed consent has been obtained from all the participants. The VTEval project was registered online at clinicaltrials.gov (identifier: NCT02156401, https://clinicaltrials.gov/ct2/show/NCT02156401).

### Data collection

The sample for the present report represents a consecutive collection of patients with suspected VTE, who were examined in the University Medical Center Mainz between April 2013 and July 2015. Within the investigation, a standardized clinical examination, a computer-assisted personal interview and venous blood sampling were performed according to standard operating procedures at the time of presentation. Information on the intake of medication was recorded digitally including assessment of ATC coding. Blood samples were collected from all participants before conduct of imaging (i.e. compression duplex ultrasound for the evaluation of the presence of DVT and CT pulmonary angiography or ventilation perfusion scintigraphy in case of suspected PE, respectively). Patients were prospectively followed-up for the incidence of arterial thrombosis (i.e. myocardial infarction, stroke or transient ischemic attack), recurrence of venous thromboembolism (i.e. deep vein thrombosis or pulmonary embolism), and all-cause death. Information on the clinical profile, intake of medication and clinical outcome over the course of time was obtained via sequential computer-assisted telephone interviews according to standard operating procedures. Information on vital status was additionally checked via governmental registration offices. All information on adverse events was validated via source data with subsequent independent adjudication. Measurements of homoarginine, ADMA and SDMA in serum samples of the study patients were conducted by LC–MS/MS according to previously described procedures^17,18^. These polar endogenous amino acids were chromatographically retained and separated by the hydrophilic-interaction-liquid-chromatography (HILIC) technique and selectively and sensitively detected by positive polarity electrospray-MS/MS analysis. For all three analytes stable isotope labeled analogs (D4-homoarginine, D7-ADMA and D6-SDMA) were applied as internal standards, leading to high precision and accuracy quantification. The relative standard deviations were 1.81% for homoarginine, 2.12% for ADMA and 2.83% for SDMA, with accuracies of 97.7%, 97.4% and 102.7% of the expected values, respectively.

### Data handling and statistical analysis

Baseline characteristics are expressed as relative and absolute frequencies. Continuous variables are reported as mean with standard deviation (SD) for normally distributed values or as median with interquartile range (IQR) where appropriate. Cumulative incidence rates were obtained from Kaplan–Meier estimates, and differences were tested by the log-rank test. The associations of selected variables with outcomes were assessed with a univariate Cox proportional hazards model, and variables that were significant in the univariate model were then entered into a multivariate Cox model using a stepwise method. Hazard ratios (HR) with the corresponding 95% confidence intervals (CI) were estimated. Comparisons of the characteristics between groups stratified for homoarginine, ADMA or SDMA levels were performed with an ANOVA test; a chi-square test was used for categorical variables. A probability value < 0.05 was considered significant. All data were statistically analyzed using R version 3.4.3^19^.

## Results

### Baseline characteristics of the patients

**Figure 1:**
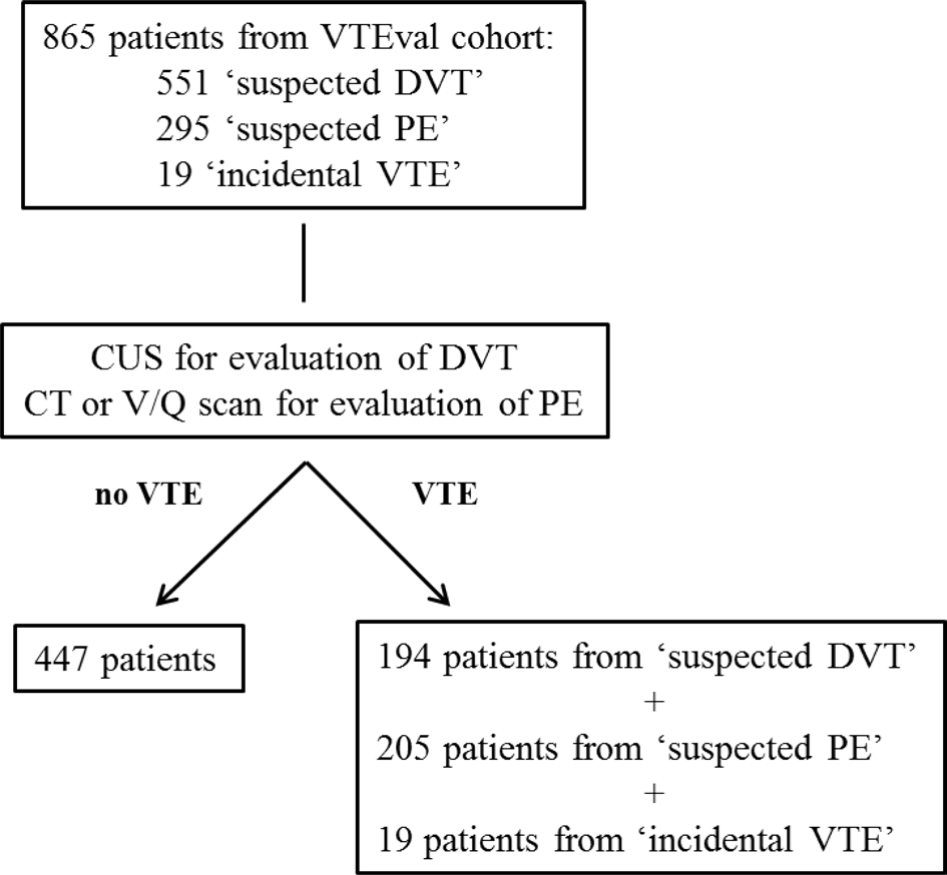
Consort flow chart. DVT, deep vein thrombosis; PE, pulmonary embolism; VTE, venous thromboembolism; CUS, compression ultrasound; V/Q scan, ventilation-perfusion scintigraphy; CT, computed tomography.

A total of 865 patients from the VTEval study cohort^16^ were included in the present analysis. Following imaging, VTE was ruled out in 447 patients and these were defined as control cohort. VTE was confirmed in 194 patients from 551 in the “suspected DVT” cohort and 205 patients from 295 in the “suspected PE” cohort. Together with the 19 cases of incidental VTE, a total of 418 patients formed the experimental cohort (Fig. 1). The average age of the study patients was 59.3 ± 16.7 years. 49.7% of the study population were males. 87.3% of the patients in the experimental cohort had DVT and 55.9% had PE. 42.5% of the VTE cases were unprovoked. 15% of the VTE patients had either active cancer or history of cancer. Additional clinical parameters of the study population are listed in the Table 1.

**Table 1:** Baseline characteristics of the patients.

Variable	All (N = 865)	Controls (N = 447)	Cases (N = 418)
**Baseline parameters**			
Sex (m)	49.7% (430/865)	41.8% (187/447)	58.1% (243/418)
Age	59.3 (16.7)	58.3 (17.5)	60.3 (15.7)
BMI	27.5 (24.0/31.5)	27.3 (24.0/32.0)	27.8 (24.1/31.0)
HR < 110 bpm	95.8% (687/717)	96.3% (366/380)	95.3% (321/337)
SBP < 100 mmHg	2.5% (17/693)	3.0% (11/371)	1.9% (6/322)
O_2_ saturation < 90%	10.1% (69/686)	6.3% (23/366)	14.4% (46/320)
DVT (yes)	43.0% (365/848)	0% (0/430)	87.3% (365/418)
PE (yes)	43.1% (233/540)	0% (0/123)	55.9% (233/417)
Origin of VTE (unprovoked)	42.5% (174/409)	N/A (0/0)	42.5% (174/409)
Active or history of cancer (yes)	11.4% (98/856)	8.1% (36/444)	15.0% (62/412)
**Clinical profile**			
Smoker (yes)	18.7% (156/835)	20.1% (87/433)	17.2% (69/402)
Obesity (yes)	33.0% (258/782)	33.9% (136/401)	32.0% (122/381)
Diabetes mellitus (yes)	12.7% (106/836)	13.1% (57/436)	12.2% (49/400)
Dyslipidemia (yes)	17.4% (143/824)	17.6% (76/432)	17.1% (67/392)
Arterial Hypertension (yes)	48.3% (405/839)	49.9% (219/439)	46.5% (186/400)
Family hist. of Mi/stroke (yes)	43.6% (349/801)	43.8% (184/420)	43.3% (165/381)
Atrial fibrillation (yes)	7.5% (61/818)	9.9% (42/424)	4.8% (19/394)
Chronic heart failure (yes)	5.5% (45/818)	6.1% (26/425)	4.8% (19/393)
Coronary artery disease (yes)	9.1% (75/827)	11.6% (50/431)	6.3% (25/396)
Peripheral artery occlusive disease (yes)	3.6% (29/815)	3.3% (14/426)	3.9% (15/389)
**Biomarkers**			
DDimer (mg/l FEU)	1.36 (0.67/3.91)	0.88 (0.51/1.55)	3.19 (1.20/6.78)
Troponin I (pg/ml)	3.55 (1.50/11.21)	2.50 (1.50/6.90)	4.80 (1.70/21.83)

*BMI* body mass index, *HR* heart rate, *SBP* systolic blood pressure, *DVT* deep vein thrombosis, *PE* pulmonary embolism, *VTE* venous thromboembolism.

### Relationship between homoarginine and clinical phenotype in the patients with VTE

The median (Q25/Q75) homoarginine concentration in the entire cohort was 1.39 (0.91/1.89) µmol/L. The plasma concentration of homoarginine was app. 10% lower in the experimental cohort, compared with the control cohort (*p* = 0.0014). At baseline low homoarginine levels were associated with increased age, presence or history of cancer, low BMI, diabetes, dyslipidemia, hypertension, atrial fibrillation, D-Dimers and Troponin I in the control cohort (Supplemental Table 1) and with female sex, increased age, unprovoked origin of VTE and presence or history of cancer in the experimental cohort (Table 2).

**Table 2:** Variables by tertiles of log(Homoarginine) in the cases only.

Variable	All (418)	Log(Homoarginine) tertile			*p* for trend
		[− 2.07,0.06] (163)	(0.06,0.51] (131)	(0.51,1.78] (124)	
**Baseline parameters**					
Sex (m)	58.1% (243/418)	49.7% (81/163)	55.0% (72/131)	72.6% (90/124)	0.00014
Age	60.3 (15.7)	64.1 (14.4)	59.9 (15.9)	55.8 (16.0)	< 0.0001
BMI	28.3 (5.6)	27.4 (5.2)	29.5 (6.1)	28.4 (5.4)	0.11
HR < 110 bpm	95.3% (321/337)	95.3% (122/128)	93.1% (95/102)	97.2% (104/107)	0.54
SBP < 100 mmHg	1.9% (6/322)	1.7% (2/119)	3.0% (3/99)	1.0% (1/104)	0.72
O_2_ saturation < 90%	14.4% (46/320)	18.0% (22/122)	11.0% (11/100)	13.3% (13/98)	0.28
DVT (yes)	87.3% (365/418)	90.7% (136/150)	87.7% (114/130)	83.3% (115/138)	0.062
PE (yes)	55.9% (233/417)	58.0% (87/150)	47.3% (61/129)	61.6% (85/138)	0.58
Origin of VTE (unprovoked)	42.5% (174/409)	36.7% (54/147)	42.5% (54/127)	48.9% (66/135)	0.039
Active or history of cancer (yes)	15.0% (62/412)	7.4% (11/148)	19.7% (25/127)	19.0% (26/137)	0.0058
**Clinical profile**					
Smoker (yes)	17.2% (69/402)	14.5% (21/145)	23.8% (30/126)	13.7% (18/131)	0.92
Obesity (yes)	32.0% (122/381)	35.0% (48/137)	30.0% (36/120)	30.6% (38/124)	0.44
Diabetes (yes)	12.2% (49/400)	7.6% (11/145)	15.3% (19/124)	14.5% (19/131)	0.074
Dyslipidemia (yes)	17.1% (67/392)	16.7% (24/144)	17.1% (21/123)	17.6% (22/125)	0.84
Hypertension (yes)	46.5% (186/400)	44.1% (64/145)	47.6% (59/124)	48.1% (63/131)	0.51
Family hist. of Mi/stroke (yes)	43.3% (165/381)	40.4% (57/141)	45.8% (54/118)	44.3% (54/122)	0.51
Atrial fibrillation (yes)	4.8% (19/394)	3.5% (5/144)	4.9% (6/123)	6.3% (8/127)	0.28
Chronic heart failure (yes)	4.8% (19/393)	2.8% (4/144)	5.7% (7/122)	6.3% (8/127)	0.17
Coronary artery disease (yes)	6.3% (25/396)	5.5% (8/146)	7.4% (9/122)	6.2% (8/128)	0.78
Peripheral artery occlusive disease (yes)	3.9% (15/389)	4.2% (6/142)	3.3% (4/123)	4.0% (5/124)	0.92
**Biomarkers**					
DDimer (mg/l FEU)	3.19 (1.20/6.78)	2.99 (1.15/6.74)	3.19 (1.29/6.80)	3.28 (1.19/6.86)	0.69
Troponin I (pg/ml)	4.80 (1.70/21.83)	4.20 (1.50/18.93)	4.30 (1.60/24.03)	6.00 (2.07/25.23)	0.17

*BMI* body mass index, *HR* heart rate, *SBP* systolic blood pressure, *DVT* deep vein thrombosis, *PE* pulmonary embolism, *VTE* venous thromboembolism.

**Figure 2:**
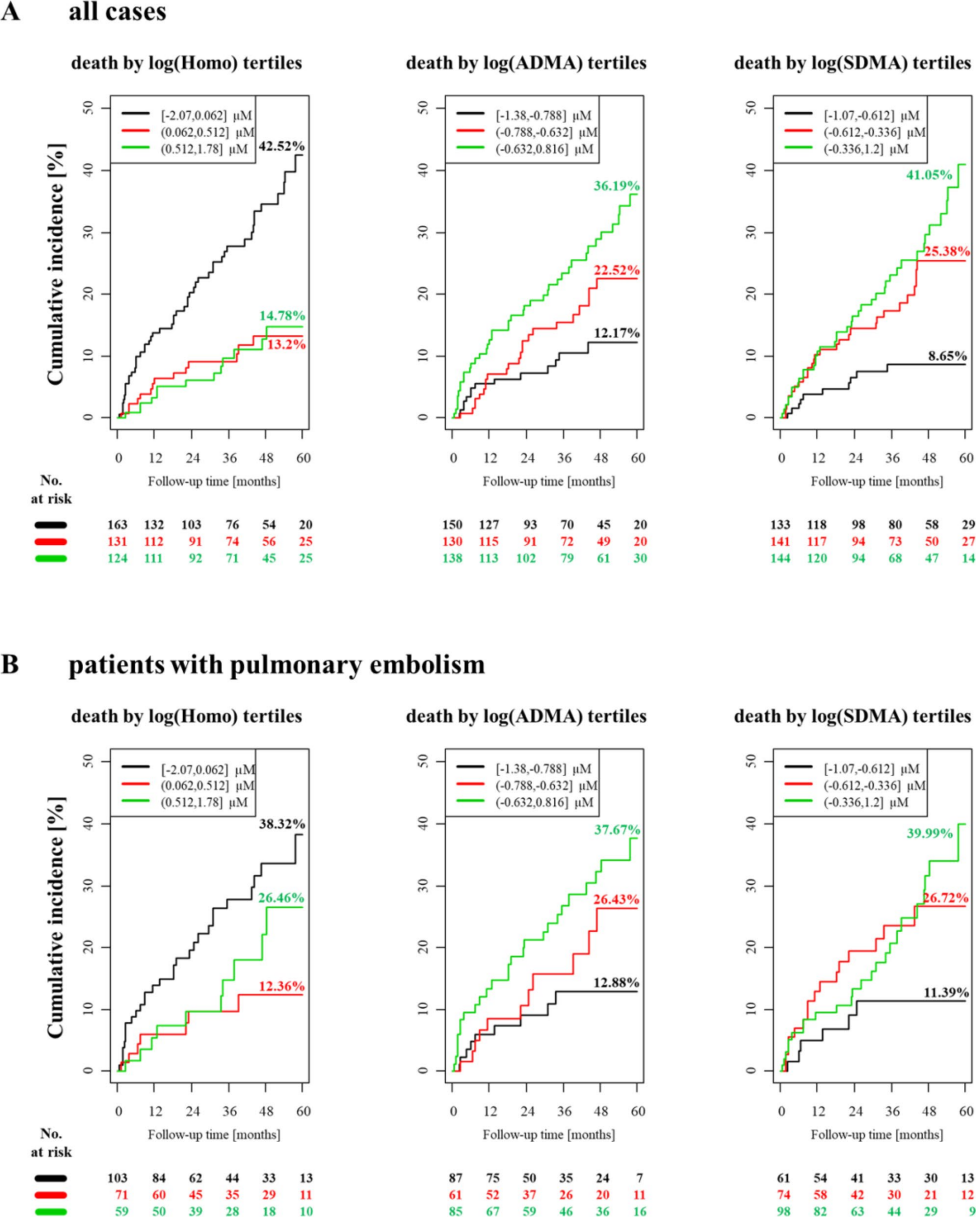

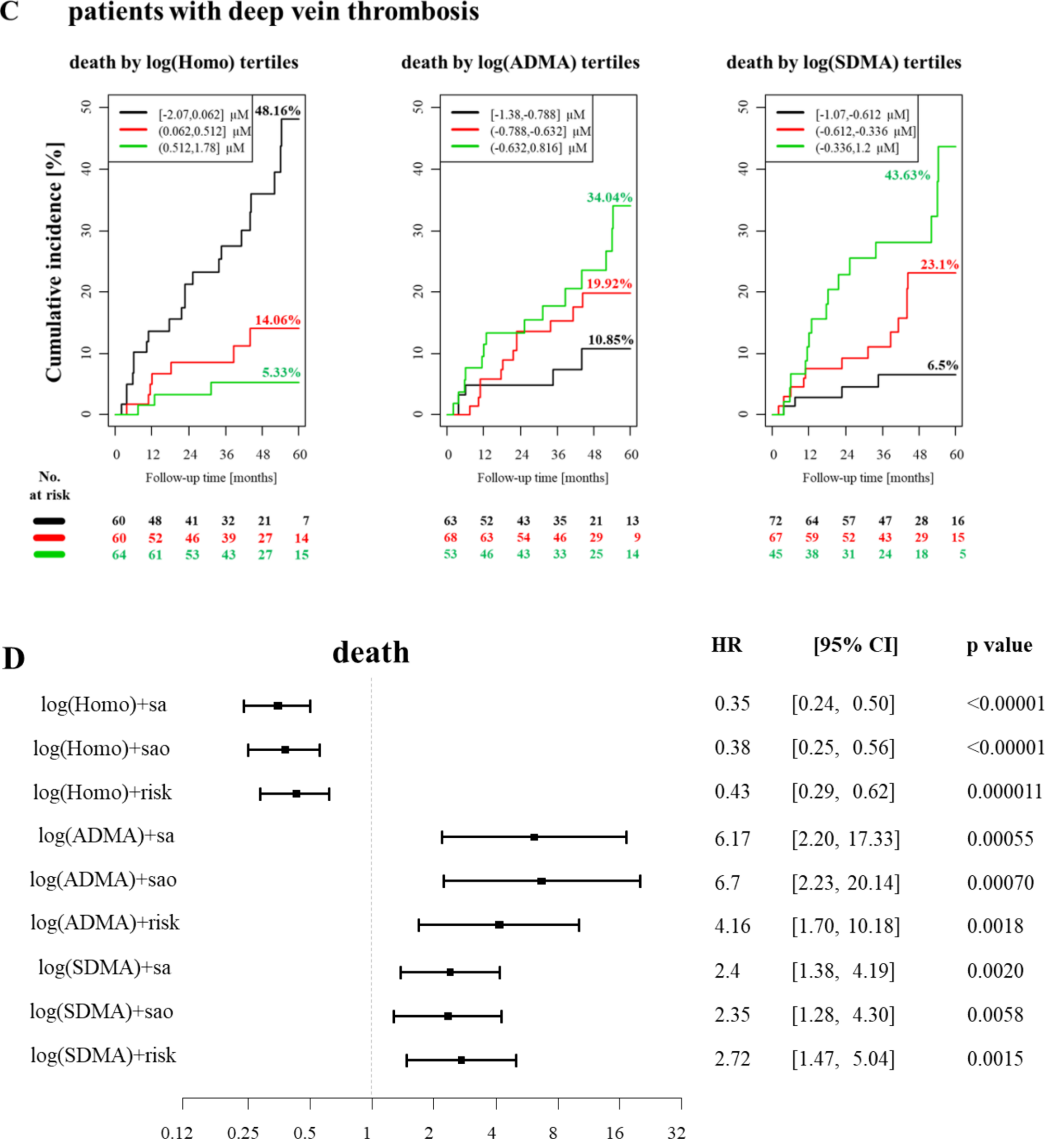
Homoarginine, ADMA, SDMA and the risk of death in the patients with confirmed VTE (cases). (**A**) Kaplan–Meier curves for all the VTE cases (**B**) Kaplan–Meier curves for all the PE cases (**C**) Kaplan–Meier curves for all the DVT cases (**D**) Cox regression analysis for all VTE cases. Adjustment for sex and age (‘sa'), sex, age and OAC (‘sao') and for sex, age, OAC, BMI, arterial hypertension, diabetes mellitus, smoking, dyslipidemia, chronic heart failure, history of stroke, creatinine and cancer (‘risk'). Homo, homoarginine; ADMA, asymmetric dimethylarginine; SDMA, symmetric dimethylarginine; VTE, venous thromboembolism; PE, pulmonary embolism; DVT, deep vein thrombosis; OAC, oral anticoagulation; BMI, body mass index; HR, hazard ratio; CI, confidence interval.

**Figure 3:**
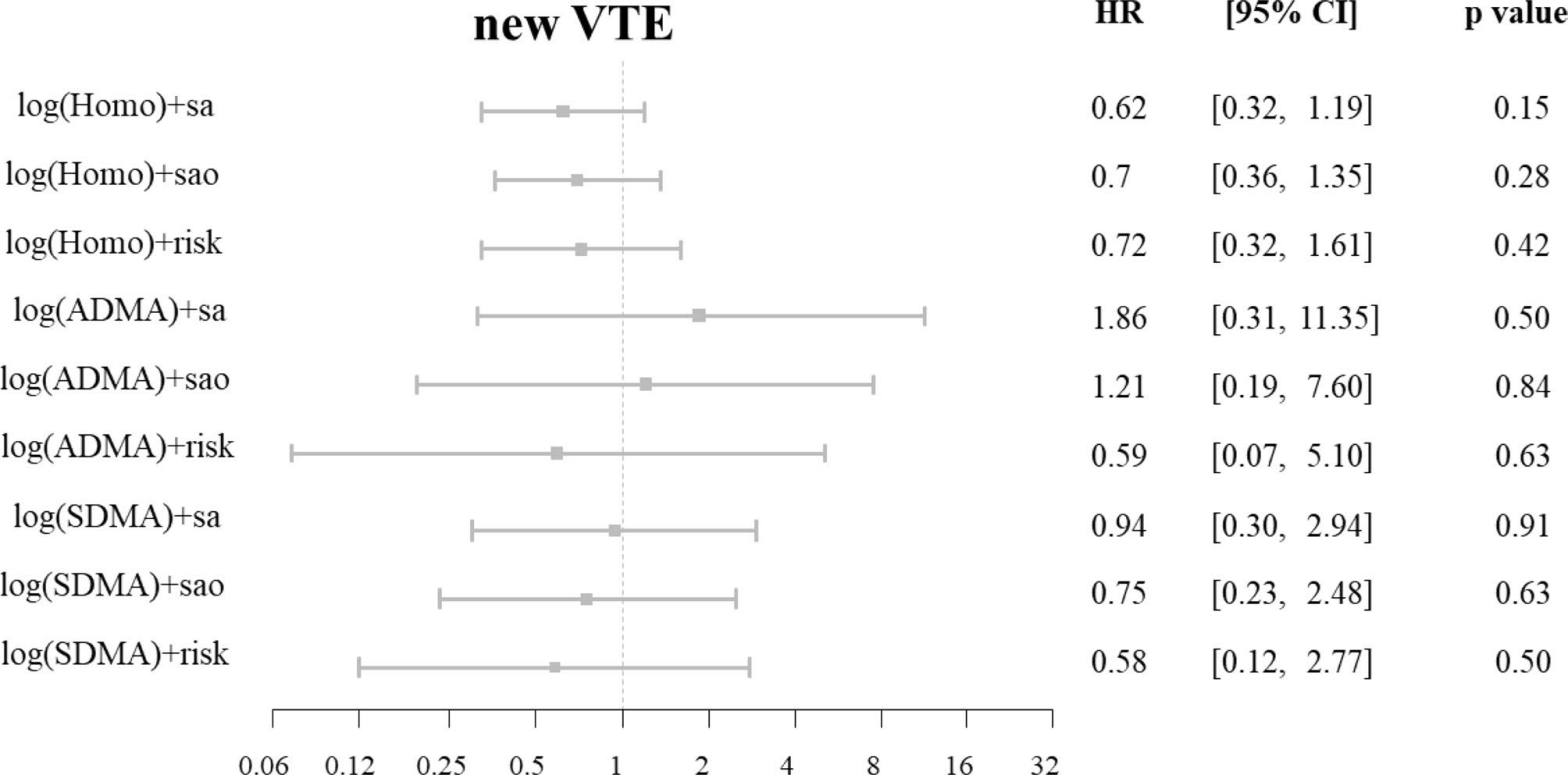
Homoarginine, ADMA, SDMA and the risk of new VTE in the patients with confirmed VTE (cases). Cox regression analysis. Adjustment for sex and age (‘sa'), sex, age and OAC (‘sao') and for sex, age, OAC, BMI, arterial hypertension, diabetes mellitus, smoking, dyslipidemia, chronic heart failure, history of stroke, creatinine and cancer (‘risk'). ‘new VTE’ encompasses recurrent DVT and/or recurrent PE. Homo, homoarginine; ADMA, asymmetric dimethylarginine; SDMA, symmetric dimethylarginine; VTE, venous thromboembolism; OAC, oral anticoagulation; BMI, body mass index; DVT, deep vein thrombosis; PE, pulmonary embolism; HR, hazard ratio; CI, confidence interval.

Next, we investigated whether the baseline plasma homoarginine concentrations can predict an unfavorable outcome in patients with confirmed VTE. The median (Q25/Q75) follow-up time for mortality was 1196 (594/1695) and for events 363 (111/1082) days. 78 patients from 418 died in the experimental cohort and 39 developed new VTE during the follow-up. After adjustment for sex, age, oral anticoagulants (OAC), body mass index (BMI), arterial hypertension, diabetes mellitus, smoking, dyslipidemia, chronic heart failure, history of stroke, creatinine and cancer, we found that low homoarginine concentrations independently predicted death (Fig. 2, Panels A and D). The correlation between low homoarginine concentrations and mortality remained statistically significant in patients with PE and those with DVT without PE (Fig. 2, Panels B and C). In contrast, we did not find any correlation between low homoarginine levels and VTE recurrence (Fig. 3).

### Relationship between methylarginines and clinical phenotype in the patients with VTE

The median (Q25/Q75) concentrations of ADMA and SDMA in the entire cohort were 0.49 (0.43/0.56) and 0.62 (0.51/0.77) µmol/L correspondingly. There was no statistically significant difference in the plasma concentration of ADMA and SDMA between the control and the experimental cohorts. At the baseline higher ADMA levels were associated with increased age, increased BMI, oxygen saturation below 90%, obesity, dyslipidemia, hypertension, atrial fibrillation, D-Dimers and Troponin I in the control cohort (Supplementary Table 2) and with older age, unprovoked origin of VTE and cancer in the experimental cohort (Table 3). Increased SDMA levels were associated with male gender, increased age, oxygen saturation below 90%, diabetes, dyslipidemia, hypertension, atrial fibrillation, coronary artery disease, D-Dimers and Troponin I in the control cohort (Supplementary Table 3) and with older age, oxygen saturation below 90% at the time of admission, presence of VTE, unprovoked origin of VTE, higher values of D-Dimer and Troponin I, presence of PE, smoking, diabetes mellitus, arterial hypertension, atrial fibrillation and chronic heart failure were associated with higher SDMA concentrations (Table 4).

**Table 3:** Variables by tertiles of log(ADMA) in the cases only.

Variable	All (418)	Log(ADMA) tertile			*p* for trend
		[− 1.38, − 0.788] (150)	(− 0.788, − 0.632] (130)	(− 0.632,0.816] (138)	
**Baseline parameters**					
Sex (m)	58.1% (243/418)	62.0% (93/150)	56.2% (73/130)	55.8% (77/138)	0.28
Age	60.3 (15.7)	56.5 (16.1)	60.1 (14.9)	64.7 (15.1)	< 0.0001
BMI	28.3 (5.6)	28.1 (5.6)	28.2 (5.3)	28.7 (5.9)	0.67
HR < 110 bpm	95.3% (321/337)	93.5% (116/124)	97.1% (102/105)	95.4% (103/108)	0.49
SBP < 100 mmHg	1.9% (6/322)	0% (0/117)	4.9% (5/103)	1.0% (1/102)	0.52
O_2_ saturation < 90%	14.4% (46/320)	13.4% (15/112)	14.6% (15/103)	15.2% (16/105)	0.70
DVT (yes)	87.3% (365/418)	90.7% (136/150)	87.7% (114/130)	83.3% (115/138)	0.062
PE (yes)	55.9% (233/417)	58.0% (87/150)	47.3% (61/129)	61.6% (85/138)	0.58
Origin (unprovoked)	42.5% (174/409)	36.7% (54/147)	42.5% (54/127)	48.9% (66/135)	0.039
Active or history of cancer (yes)	15.0% (62/412)	7.4% (11/148)	19.7% (25/127)	19.0% (26/137)	0.0058
**Clinical profile**					
Smoker (yes)	17.2% (69/402)	14.5% (21/145)	23.8% (30/126)	13.7% (18/131)	0.92
Obesity (yes)	32.0% (122/381)	35.0% (48/137)	30.0% (36/120)	30.6% (38/124)	0.44
Diabetes (yes)	12.2% (49/400)	7.6% (11/145)	15.3% (19/124)	14.5% (19/131)	0.074
Dyslipidemia (yes)	17.1% (67/392)	16.7% (24/144)	17.1% (21/123)	17.6% (22/125)	0.84
Hypertension (yes)	46.5% (186/400)	44.1% (64/145)	47.6% (59/124)	48.1% (63/131)	0.51
Family hist. of Mi/stroke (yes)	43.3% (165/381)	40.4% (57/141)	45.8% (54/118)	44.3% (54/122)	0.51
Atrial fibrillation (yes)	4.8% (19/394)	3.5% (5/144)	4.9% (6/123)	6.3% (8/127)	0.28
Chronic heart failure (yes)	4.8% (19/393)	2.8% (4/144)	5.7% (7/122)	6.3% (8/127)	0.17
Coronary artery disease (yes)	6.3% (25/396)	5.5% (8/146)	7.4% (9/122)	6.2% (8/128)	0.78
Peripheral artery occlusive disease (yes)	3.9% (15/389)	4.2% (6/142)	3.3% (4/123)	4.0% (5/124)	0.92
**Biomarkers**					
DDimer (mg/l FEU)	3.19 (1.20/6.78)	2.99 (1.15/6.74)	3.19 (1.29/6.80)	3.28 (1.19/6.86)	0.69
Troponin I (pg/ml)	4.80 (1.70/21.83)	4.20 (1.50/18.93)	4.30 (1.60/24.03)	6.00 (2.07/25.23)	0.17

*BMI* body mass index, *HR* heart rate, *SBP* systolic blood pressure, *DVT* deep vein thrombosis, *PE* pulmonary embolism, *VTE* venous thromboembolism.

**Table 4:** Variables by tertiles of log(SDMA) in the cases only.

Variable	All (418)	Log(SDMA) tertile			*p* for trend
		[− 1.07, − 0.61] (133)	(− 0.61, − 0.33] (141)	(− 0.33,1.2] (144)	
**Baseline parameters**					
Sex (m)	58.1% (243/418)	55.6% (74/133)	61.7% (87/141)	56.9% (82/144)	0.84
Age	60.3 (15.7)	51.9 (15.5)	60.0 (14.5)	68.5 (12.7)	< 0.0001
BMI	28.3 (5.6)	28.1 (5.7)	28.8 (6.1)	28.1 (4.9)	0.79
HR < 110 bpm	95.3% (321/337)	97.2% (103/106)	92.4% (110/119)	96.4% (108/112)	0.82
SBP < 100 mmHg	1.9% (6/322)	1.9% (2/107)	3.6% (4/112)	0% (0/103)	0.33
O_2_ saturation < 90%	14.4% (46/320)	11.5% (12/104)	9.7% (11/113)	22.3% (23/103)	0.027
DVT (yes)	87.3% (365/418)	94.0% (125/133)	86.5% (122/141)	81.9% (118/144)	0.0027
PE (yes)	55.9% (233/417)	45.9% (61/133)	52.5% (74/141)	68.5% (98/143)	0.00014
Origin (unprovoked)	42.5% (174/409)	35.4% (46/130)	44.2% (61/138)	47.5% (67/141)	0.045
Active or history of cancer (yes)	15.0% (62/412)	8.4% (11/131)	16.7% (23/138)	19.6% (28/143)	0.010
**Cardiovascular risk factors**					
Smoker (yes)	17.2% (69/402)	20.9% (27/129)	22.0% (29/132)	9.2% (13/141)	0.0095
Obesity (yes)	32.0% (122/381)	32.2% (39/121)	34.1% (43/126)	29.9% (40/134)	0.67
Diabetes (yes)	12.2% (49/400)	4.7% (6/127)	13.6% (18/132)	17.7% (25/141)	0.0013
Dyslipidemia (yes)	17.1% (67/392)	12.7% (16/126)	16.4% (21/128)	21.7% (30/138)	0.051
Hypertension (yes)	46.5% (186/400)	39.4% (50/127)	43.6% (58/133)	55.7% (78/140)	0.0070
Family hist. of Mi/stroke (yes)	43.3% (165/381)	37.6% (47/125)	47.2% (58/123)	45.1% (60/133)	0.23
Atrial fibrillation (yes)	4.8% (19/394)	0.8% (1/127)	6.2% (8/128)	7.2% (10/139)	0.016
Chronic heart failure (yes)	4.8% (19/393)	0.8% (1/124)	3.1% (4/129)	10.0% (14/140)	0.00044
Coronary artery disease (yes)	6.3% (25/396)	1.6% (2/124)	9.8% (13/132)	7.1% (10/140)	0.077
Peripheral artery occlusive disease (yes)	3.9% (15/389)	2.4% (3/124)	3.9% (5/128)	5.1% (7/137)	0.26
**Biomarkers**					
DDimer (mg/l FEU)	3.19 (1.20/6.78)	2.27 (0.96/6.62)	2.93 (1.22/6.76)	3.99 (1.34/7.36)	0.034
Troponin I (pg/ml)	4.80 (1.70/21.83)	2.40 (1.50/7.33)	4.20 (1.60/15.71)	13.60 (3.98/52.08)	< 0.0001

*BMI* body mass index, *HR* heart rate, *SBP* systolic blood pressure, *DVT* deep vein thrombosis, *PE* pulmonary embolism, *VTE* venous thromboembolism.

After adjustments for sex, age, oral anticoagulants (OAC), body mass index (BMI), arterial hypertension, diabetes mellitus, smoking, dyslipidemia, chronic heart failure, history of stroke, creatinine and cancer, we found that both methylarginines were independent predictors for death, but not for recurrent VTE alone (Fig. 2, Panel A). The correlation between high methylarginines levels and mortality remained statistically significant, when the patients with PE and the patients with DVT without PE were analyzed separately (Fig. 2, Panels B and C). There was no correlation between high levels of methylarginines and recurrence of VTE (Fig. 3).

### Low homoarginine and high methylarginines as outcome predictors in the patients without VTE

**Figure 4:**
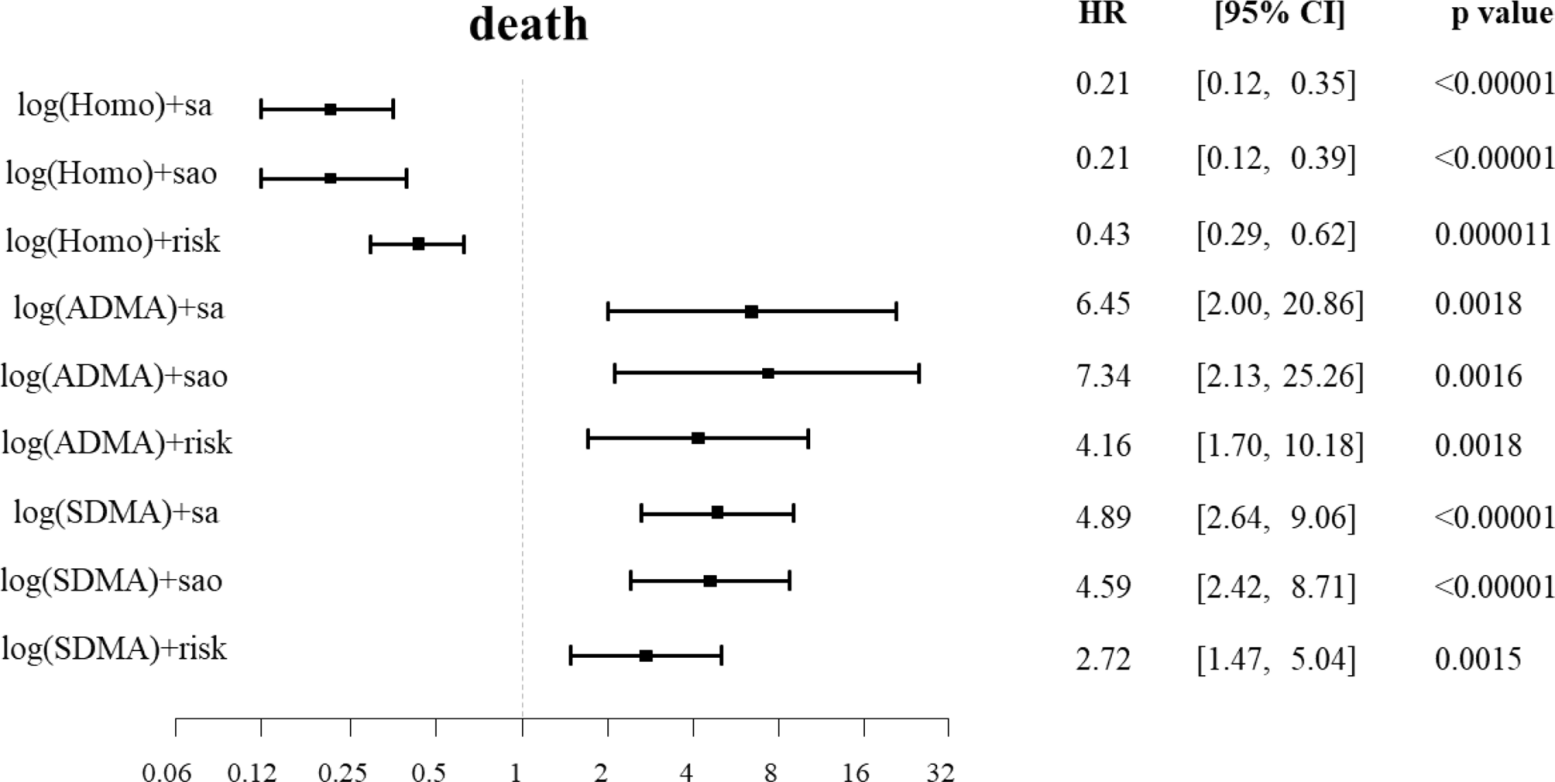
Homoarginine, ADMA, SDMA and the risk of death in the patients with ruled out VTE (controls). Cox regression analysis. Adjustment for sex and age (‘sa'), sex, age and OAC (‘sao') and for sex, age, OAC, BMI, arterial hypertension, diabetes mellitus, smoking, dyslipidemia, chronic heart failure, history of stroke, creatinine and cancer (‘risk'). Homo, homoarginine; ADMA, asymmetric dimethylarginine; SDMA, symmetric dimethylarginine; BMI, body mass index; HR, hazard ratio; CI, confidence interval.

Interestingly, we also observed correlations between low homoarginine or high methylarginines and death in the patients without VTE. However, there was no correlation between these markers and the development of recurrent VTE (Fig. 4).

## Discussion

The main findings of the current manuscript are: (1) low serum homoarginine levels independently predict all-cause mortality in VTE patients over long-term follow-up without correlating with VTE recurrence; (2) elevated serum levels of asymmetric dimethylarginine (ADMA) and symmetric dimethylarginine (SDMA) are independent predictors of death after VTE; (3) low homoarginine and high methylarginines also predict death in the patients with clinical suspicion of VTE, in whom VTE has been ruled out.

In our study, we observed previously demonstrated associations between low homoarginine levels at baseline in the control group and increased age, presence or history of cancer, diabetes, dyslipidemia, hypertension, atrial fibrillation and D-Dimers^8^ as well as associations between increased ADMA at baseline and increased age, obesity, dyslipidemia, hypertension, atrial fibrillation, D-Dimers and between increased SDMA levels at baseline and increased age, diabetes, dyslipidemia, hypertension, atrial fibrillation, coronary artery disease, D-Dimers and Troponin I^7^.

The following baseline characteristics were different between the control and cases groups: homoarginine levels (lower in cases), sex (more males in cases), prevalence of cancer (higher in cases), prevalence of atrial fibrillation (lower in cases), prevalence of coronary artery disease (lower in cases), D-Dimers (higher in cases), Troponin I (higher in cases) and prevalence of patients with O_2_ saturation less than 90% (higher in cases). The fact that we observed lower homoarginine levels in the patients with VTE, while we did not see any statistically significant association between low homoarginine levels and the risk of VTE recurrence, is suggestive that homoarginine is not a prothrombotic marker, but rather a general marker of “frailty”, with more “frail” patients being less mobile and having more comorbidities at the baseline, which would explain, why those patients would be at higher risk for VTE. The higher prevalence of male sex among the patients with VTE in the cohort with the average age of 59.3 years is consistent with the previous observation that incidence of VTE at younger age is higher in women and at older age higher in men^20^. The association between cancer and VTE is also well known^21^. Lower prevalence of atrial fibrillation and coronary artery disease in patients with VTE might be explained by more common usage of anticoagulants and anti-platelet agents in patients with those diseases. D-Dimers are the classic marker of VTE, while Troponin I is a biomarker of acute right heart failure in patients with PE. Higher prevalence of hypoxemia in cases can be explained by PE.

One of the key findings in the current study is that low homoarginine levels independently predicted long-term mortality in VTE patients. These results are in line with the recent discovery that low homoarginine levels correlate with all-cause mortality in treatment-naive patients with pulmonary arterial hypertension^22^. In that study low homoarginine levels also correlated with the severity of pulmonary arterial hypertension as assessed by NT-proBNP, cardiac output, right atrial pressure and six minute walking distance^22^. Interestingly, low homoarginine levels are also predictive of all-cause mortality in the patients with left heart failure^23,24^. Our current findings are also consistent with the previous studies, which demonstrated that low homoarginine levels independently predict mortality in patients referred for coronary angiography^12,14^, in patients with heart failure^24^, with peripheral artery disease^25^, stroke^13^, in elderly individuals^26^ and in general population^27^. It will have to be determined by the future interventional studies with homoarginine supplementation, whether homoarginine is merely a marker of increased mortality in the VTE patients or whether it serves direct protective role, e.g. by protecting from myocardial remodeling in the settings of VTE-induced chronic pulmonary hypertension. The second option would be in line with the experimental observations by us and others that homoarginine supplementation protects from myocardial remodeling in animal models of heart failure^28–30^.

Another important finding in the present study is that higher concentrations of ADMA and SDMA are associated with increased mortality in VTE patients. These results are in agreement with the previous report that circulating ADMA concentrations are increased in chronic thromboembolic pulmonary hypertension^31^ and that circulating concentrations of ADMA and SDMA are elevated in pulmonary arterial hypertension^32^.

Furthermore, our results are consisted with the previous reports on association between high serum levels of dimethylarginines and increased mortality in patients with stable and unstable ischemic heart disease (ADMA)^33^, in patients with end-stage renal disease (ADMA)^34^, in patients after ischemic stroke (SDMA)^35^ and in general population (SDMA)^36^. We observed neither association between ADMA and SDMA levels and VTE at the baseline, nor association between those metabolites and VTE recurrence. This would be consistent with the findings of Haider et al., who saw no difference in the levels of ADMA and SDMA between subjects with and without DVT^37^. Our findings that in VTE patients SDMA serum levels are associated with the presence of PE and inversely correlated with the presence of DVT might be suggestive that increased SDMA levels could be a marker of embolization in patients with DVT. Our observation that low homoarginine levels and high methylarginines levels were not specific to VTE as independent predictors of mortality in the investigated patient cohort but also predicted mortality in the control group of patients raises the possibility that these compounds might be independent markers of frailty and mortality independently of the presence of VTE. This would be consistent with the finding that neither low homoarginine nor high methylarginines predicted recurrence of VTE. In order to answer the question how VTE is affected by low homoarginine and high methylarginines one would have to look at a large population and assess the rate of VTE in patients with and without low levels of homoarginine or with and without high methylarginines.

### Strengths and limitations

The strength of this study is that it is the first demonstration that low homoarginine and high methylarginines independently predict long-term outcome in patients presenting with suspicion of venous thromboembolism.

A limitation of the current study is that the number of the patients in “incidental VTE” group was low, so no conclusions can be drawn for this subgroup. Secondly, it remains unclear whether homoarginine and methylarginines are an overall marker of poor long term prognosis, or whether they actually contribute to long term death and, if yes, than by which mechanism, since VTE recurrence does not seem to be affected. Furthermore, it would have been very important to distinguish the causes of death in the current study, but these data are unfortunately not available.

### Potential diagnostic and therapeutic applications

This study suggests several directions for improvement of risk assessment and treatment of the patients with VTE in the future. First,a possible clinical significance of our findings might be that, even if low serum homoarginine levels and high serum dimethylarginine levels are nonspecific to VTE, they may still be used for risk stratification in the clinical population of VTE patients, similarly to some other risk factors, which are nonspecific to VTE, such as age, sex, history of cancer, history of heart failure, history of chronic pulmonary diseases etc., and still are used for risk stratification in patients with pulmonary embolism. It will have to be investigated in the future studies, whether addition of homoarginine and dimethylarginines as biomarkers for risk stratification in the VTE population on the top of the already available biomarkers would result in better risk assessment and would lead to significant clinical benefits. Second, an attractive therapeutic approach might be to supplement homoarginine in patients with acute VTE and high clot burden. Indeed, the safety of homoarginine supplementation has been demonstrated in the Phase I clinical study in healthy volunteers^38^, while the Phase II study in another clinical population, i.e. in the patients with ischemic stroke, is ongoing (ClinicalTrials.gov Identifier: NCT03692234). Another possible approach would be to develop therapeutic interventions aiming at lowering ADMA and SDMA, by e.g. upregulating the enzymes DDAHs and AGXT2, which are involved into their metabolism, however, this approach, even though it should also be pursued, is much more challenging than the straightforward dietary homoarginine supplementation.

## Supplementary Information

Supplementary Information 1.
