# Supplementary material for: Homoarginine and methylarginines independently predict long-term outcome in patients presenting with suspicion of venous thromboembolism
Source: Sci Rep. 2021 May 5;11:9569. doi: 10.1038/s41598-021-88986-y (PMC8100302; doi:10.1038/s41598-021-88986-y)
Supplement: Supplementary file 1 — Supplementary Information 1. [file 41598_2021_88986_MOESM1_ESM.pdf]

Supplementary Material

Supplemental Table 1. Variables by tertiles of log(Homoarginine) in the controls only.

Variable	All (447)	Log(Homoarginine) tertile			p for trend
		[-2.07,0.06] (126)	(0.06,0.51] (157)	(0.51,1.78] (164)	
**Baseline parameters**					
Sex (m)	41.8% (187/447)	42.1% (53/126)	36.3% (57/157)	47.0% (77/164)	0.33
Age	58.3 (17.5)	65.1 (16.0)	58.3 (16.3)	53.0 (18.0)	<0.0001
BMI	27.3 (24.0/32.0)	26.5 (23.2/30.3)	27.5 (24.6/31.8)	28.0 (24.3/32.8)	0.026
HR <110 bpm	96.3% (366/380)	92.8% (103/111)	99.2% (128/129)	96.4% (135/140)	0.17
SBP <100 mmHg	3.0% (11/371)	4.7% (5/106)	2.3% (3/129)	2.2% (3/136)	0.27
O_2 saturation <90%	6.3% (23/366)	9.0% (9/100)	7.7% (10/130)	2.9% (4/136)	0.050
Active or history of cancer (yes)	8.1% (36/444)	14.4% (18/125)	5.8% (9/156)	5.5% (9/163)	0.0087
**Clinical profile**					
Smoker (yes)	20.1% (87/433)	15.8% (19/120)	19.6% (30/153)	23.8% (38/160)	0.10
Obesity (yes)	33.9% (136/401)	27.6% (32/116)	33.3% (47/141)	39.6% (57/144)	0.041
Diabetes (yes)	13.1% (57/436)	19.8% (24/121)	9.0% (14/155)	11.9% (19/160)	0.072
Dyslipidemia (yes)	17.6% (76/432)	23.3% (28/120)	18.3% (28/153)	12.6% (20/159)	0.019
Hypertension (yes)	49.9% (219/439)	58.2% (71/122)	49.7% (77/155)	43.8% (71/162)	0.017
Family hist. of Mi/stroke (yes)	43.8% (184/420)	43.9% (50/114)	43.6% (65/149)	43.9% (69/157)	0.98
Atrial fibrillation (yes)	9.9% (42/424)	22.2% (26/117)	6.0% (9/149)	4.4% (7/158)	<0.0001
Chronic heart failure (yes)	6.1% (26/425)	11.6% (14/121)	2.0% (3/147)	5.7% (9/157)	0.070
Coronary artery disease (yes)	11.6% (50/431)	17.8% (21/118)	7.9% (12/152)	10.6% (17/161)	0.090
Peripheral artery occlusive disease (yes)	3.3% (14/426)	5.1% (6/117)	2.0% (3/151)	3.2% (5/158)	0.42
**Biomarkers**					
DDimer (mg/l FEU)	0.88 (0.51/1.55)	1.18 (0.68/2.64)	0.82 (0.46/1.41)	0.72 (0.46/1.27)	<0.0001
Troponin I (pg/ml)	2.50 (1.50/6.90)	6.40 (2.04/18.61)	2.00 (1.50/5.21)	1.60 (1.50/3.83)	<0.0001

BMI, body mass index; HR, heart rate; SBP, systolic blood pressure, DVT, deep vein thrombosis; PE, pulmonary embolism; VTE, venous thromboembolism.

Supplemental Table 2. Variables by tertiles of log(ADMA) in the controls only.

Variable	All (447)	Log(ADMA) tertile			p for trend
		[-1.38,-0.788] (150)	(-0.788,-0.632] (130)	(-0.632,0.816] (138)	
**Baseline parameters**					
Sex (m)	41.8% (187/447)	41.0% (57/139)	38.6% (61/158)	46.0% (69/150)	0.38
Age	58.3 (17.5)	53.8 (18.2)	58.6 (17.2)	62.0 (16.3)	<0.0001
BMI	27.3 (24.0/32.0)	25.8 (23.0/28.9)	28.0 (24.0/32.2)	28.5 (24.4/33.3)	0.00053
HR <110 bpm	96.3% (366/380)	95.8% (115/120)	95.5% (128/134)	97.6% (123/126)	0.45
SBP <100 mmHg	3.0% (11/371)	5.9% (7/119)	0% (0/130)	3.3% (4/122)	0.24
O_2 saturation <90%	6.3% (23/366)	1.8% (2/112)	8.3% (11/132)	8.2% (10/122)	0.047
Active or history of cancer (yes)	8.1% (36/444)	7.2% (10/138)	5.1% (8/158)	12.2% (18/148)	0.12
**Clinical profile**					
Smoker (yes)	20.1% (87/433)	24.4% (33/135)	18.7% (29/155)	17.5% (25/143)	0.15
Obesity (yes)	33.9% (136/401)	21.8% (26/119)	37.1% (52/140)	40.8% (58/142)	0.0015
Diabetes (yes)	13.1% (57/436)	13.9% (19/137)	9.6% (15/157)	16.2% (23/142)	0.55
Dyslipidemia (yes)	17.6% (76/432)	14.8% (20/135)	14.0% (22/157)	24.3% (34/140)	0.038
Hypertension (yes)	49.9% (219/439)	40.1% (55/137)	49.7% (78/157)	59.3% (86/145)	0.0013
Family hist. of Mi/stroke (yes)	43.8% (184/420)	39.3% (53/135)	45.1% (69/153)	47.0% (62/132)	0.20
Atrial fibrillation (yes)	9.9% (42/424)	4.4% (6/136)	9.2% (14/152)	16.2% (22/136)	0.0012
Chronic heart failure (yes)	6.1% (26/425)	4.4% (6/136)	5.9% (9/152)	8.0% (11/137)	0.21
Coronary artery disease (yes)	11.6% (50/431)	8.8% (12/136)	9.8% (15/153)	16.2% (23/142)	0.054
Peripheral artery occlusive disease (yes)	3.3% (14/426)	2.2% (3/136)	2.6% (4/152)	5.1% (7/138)	0.18
**Biomarkers**					
DDimer (mg/l FEU)	0.88 (0.51/1.55)	0.70 (0.44/1.27)	0.91 (0.55/1.47)	1.10 (0.59/2.17)	0.00028
Troponin I (pg/ml)	2.50 (1.50/6.90)	1.50 (1.50/6.23)	2.50 (1.50/5.60)	3.10 (1.50/9.20)	0.0022

BMI, body mass index; HR, heart rate; SBP, systolic blood pressure, DVT, deep vein thrombosis; PE, pulmonary embolism; VTE, venous thromboembolism.

Supplemental Table 3. Variables by tertiles of log(SDMA) in the controls only.

Variable	All (447)	Log(SDMA) tertile			p for trend
		[-1.07,-0.61] (156)	(-0.61,-0.33] (147)	(-0.33,1.2] (144)	
**Baseline parameters**					
Sex (m)	41.8% (187/447)	30.8% (48/156)	42.2% (62/147)	53.5% (77/144)	<0.0001
Age	58.3 (17.5)	50.7 (16.3)	57.2 (17.2)	67.6 (14.6)	<0.0001
BMI	27.3 (24.0/32.0)	26.4 (23.7/31.2)	28.4 (24.0/32.5)	27.0 (24.2/31.6)	0.55
HR <110 bpm	96.3% (366/380)	97.0% (129/133)	100.0% (125/125)	91.8% (112/122)	0.033
SBP <100 mmHg	3.0% (11/371)	3.7% (5/134)	2.5% (3/120)	2.6% (3/117)	0.58
O_2 saturation <90%	6.3% (23/366)	2.3% (3/133)	5.0% (6/119)	12.3% (14/114)	0.0014
Active or history of cancer (yes)	8.1% (36/444)	5.8% (9/156)	6.8% (10/146)	12.0% (17/142)	0.052
**Cardiovascular risk factors**					
Smoker (yes)	20.1% (87/433)	26.1% (40/153)	18.9% (27/143)	14.6% (20/137)	0.014
Obesity (yes)	33.9% (136/401)	31.6% (43/136)	39.2% (51/130)	31.1% (42/135)	0.93
Diabetes (yes)	13.1% (57/436)	11.6% (18/155)	7.7% (11/142)	20.1% (28/139)	0.036
Dyslipidemia (yes)	17.6% (76/432)	13.0% (20/154)	12.1% (17/141)	28.5% (39/137)	0.00070
Hypertension (yes)	49.9% (219/439)	36.1% (56/155)	44.1% (63/143)	70.9% (100/141)	<0.0001
Family hist. of Mi/stroke (yes)	43.8% (184/420)	41.7% (63/151)	47.5% (66/139)	42.3% (55/130)	0.89
Atrial fibrillation (yes)	9.9% (42/424)	4.0% (6/151)	2.9% (4/139)	23.9% (32/134)	<0.0001
Chronic heart failure (yes)	6.1% (26/425)	4.0% (6/149)	5.0% (7/139)	9.5% (13/137)	0.056
Coronary artery disease (yes)	11.6% (50/431)	6.6% (10/151)	7.1% (10/141)	21.6% (30/139)	<0.0001
Peripheral artery occlusive disease (yes)	3.3% (14/426)	2.0% (3/152)	4.3% (6/141)	3.8% (5/133)	0.38
**Biomarkers**					
DDimer (mg/l FEU)	0.88 (0.51/1.55)	0.68 (0.41/1.13)	0.79 (0.49/1.41)	1.22 (0.71/2.52)	<0.0001
Troponin I (pg/ml)	2.50 (1.50/6.90)	1.50 (1.50/3.40)	2.00 (1.50/5.00)	6.40 (2.49/22.23)	<0.0001

BMI, body mass index; HR, heart rate; SBP, systolic blood pressure, DVT, deep vein thrombosis; PE, pulmonary embolism; VTE, venous thromboembolism.
